# Nestorone^®^, a 19nor‐progesterone derivative boosts remyelination in an animal model of demyelination

**DOI:** 10.1111/cns.13538

**Published:** 2020-12-24

**Authors:** Martine El‐Etr, Yvette Akwa, Marion Rame, Michael Schumacher, Regine Sitruk‐Ware

**Affiliations:** ^1^ Disease and Hormones of the Nervous System” U1195 Inserm‐Université Paris Saclay Le Kremlin‐Bicêtre France; ^2^ Population Council and Rockefeller University New York NY USA

**Keywords:** estradiol, multiple sclerosis, nestorone, oligodendrocytes, remyelination

## Abstract

**Introduction:**

We previously showed that Nestorone^®^ (NES), a synthetic progestin structurally related to progesterone, stimulated remyelination of the corpus callosum in a Cuprizone (CUP) mouse model of demyelination in intact females by promoting replenishment with mature oligodendrocytes (OL) (Glia. 2015;63:104‐117). Here, we further investigated the underlying mechanisms of this promyelinating effect.

**Methods:**

We explored whether NES, applied subcutaneously through Alzet mini‐osmotic pumps, regulates specific transcription factors involved in oligodendrocyte progenitor cell (OPC) proliferation and their differentiation into mature OL, using RT‐qPCR and Western Blot analysis.

**Results:**

Our present data show that in comparison to controls, a one‐week treatment with NES, through Alzet mini‐osmotic pumps, enhanced the production of three relevant transcription factor mRNAs encoding Olig2, Myt1, and Sox17. After 3 weeks, NES treatment reversed the effect of CUP on the levels of corresponding Olig2, Myt1, and Sox17 proteins. Moreover, in mice receiving NES + Estradiol (E2) co‐treatment, levels of Olig2, Myt1, and Sox17 proteins did not change as compared to NES alone.

**Conclusion:**

NES alone or with E2 increased the levels of transcription factors, essential for myelin synthesis.

## Graphical abstract

Schematic view of the first demonstration of the mechanism by which Nestorone® a synthetic progestin structurally related to progesterone stimulates remyelination of the corpus callosum in a mouse model of demyelination, by increasing the levels of transcription factors Olig2, Myt1, and Sox17 proteins, essential for myelin synthesis after one week of treatment.

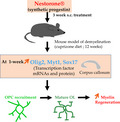

## 1 INTRODUCTION

Although spontaneous remyelination is observed in multiple sclerosis (MS), with time the physiological repair mechanism is impaired and leads to axonal degeneration with ensuing persistent functional deficits. Dysfunction and loss of OL and reduced generation of OL progenitors lead to persistent demyelination and insufficient remyelination. Therefore, there is a need to understand mechanisms of remyelination and causes of remyelination failure to be able to better design strategies for enhancing remyelination for MS therapy.

We have previously shown that progesterone (P) and its 19 nor‐derivative, Nestorone (NES), also known as segesterone acetate, a highly selective agonist to P receptors were able to promote the regeneration of myelin sheets.^1^ E2 was also shown to modulate progestin receptor levels in rodent brain regions ^2^ and combined P and E2 treatment fully protect from cuprizone‐induced demyelination in the corpus callosum of male mice.^3^ Estrogens significantly decreased disease severity in the experimental autoimmune encephalomyelitis (EAE) mouse model^4^, ^5^ and were proposed as a therapy for women with MS.^6^ Therefore, we assessed the effect of E2 combined with NES.

P or NES was administered to female mice after 12‐week feeding with the copper chelator CUP. After such a long‐term exposure to this agent, which is toxic to OL, spontaneous remyelination no longer takes place for several weeks. However, after removal of CUP from the diet, P implant or NES administered subcutaneously for 3 weeks, *via* Alzet mini‐osmotic pumps, alone or combined with E2, were able to enhance the number of mature OL and their progenitors OPC and induce remyelination. In order to elucidate the mechanism involved in this promyelinating effect of NES alone and combined with E2, we studied the expression of transcription factors known to be involved in myelin synthesis.

According to various studies performed during development and after adult demyelination, we selected 3 transcription factors involved in myelin production: (1) Myt1 a zinc finger DNA binding protein which regulates OPC differentiation into mature OL and binds to the promoter region of Proteolipid Protein (PLP), the most important myelin gene in the central nervous system (CNS)^7^; (2) Olig2, which is known to induce OPC migration and differentiation into mature OL after demyelination^8^ and (3) Sox17 which is primarily detected in newly generated and maturing OL and is important for OPC differentiation and remyelination.^9^

## 2 MATERIAL AND METHODS

### 2.1 Animals

Eight‐week‐old intact C57/Bl6 female mice were fed for 12 weeks with a powder meal containing (or not) 0.2% of the copper chelator CUP. After 12 weeks, CUP was removed from the diet and mice were treated or not (CONTROL) for 3 weeks, with 8 µg NES/d with or without 1 µg E2/d. Both steroids were solubilized in 40% Molecusol (2‐OH‐propyl‐β‐cyclodextrin). NES was administered through mini‐osmotic Alzet pumps (model # 2004) with a release rate of 0.25 µL/h. The number of animals included in the analysis of transcription factor mRNAs was per group: n = 6 for Olig2, n = 6‐8 for Myt1, and n = 6‐7 for Sox17. In the transcription factor protein analysis, the number of animals was for Olig2 n = 7‐8, Myt1 n = 3‐5, and Sox17 n = 3 per group. All experimental procedures were approved by the BEA (Bureau d’Expérimentation Animale) of the French National Institute for Health and Medical Research (Inserm).

### 2.2 RT‐qPCR analysis

Total RNA was extracted from blocks of corpus callosum/hippocampus of 1‐week‐treated animals or controls, using the Trizol Technique (Life technologies, Invitrogen) and RNeasy mini‐kits 250 (Quiagen). Reverse transcription was performed using Superscript II reverse transcriptase and dNTP set (Invitrogen), Random primers and RNasin (Promega) and followed by qPCR using the TaqMan Gene Expression Assays: PCR master Mix, predesigned Taqman primers for Myt1, Olig2 and Sox17, and endogenous control (GAPDH), Thermo‐Fast 96 and ABgene plates (Applied Biosystems, Thermofisher). Expression of genes was analyzed with the 7300 Systems SDS Software (Applied Biosystem) and the expression of Myt1, Olig2, and Sox17 normalized to reference gene GAPDH whose expression was stable in different groups.

### 2.3 Western blot

Proteins were extracted from blocks of corpus callosum/hippocampus of 3‐week‐treated animals or controls, submitted to a 100°C denaturation and solubilized in sodium dodecyl sulfate (SDS)‐PAGE sample buffer. 50 μg proteins were then added to wells containing SDS‐PAGE gel and submitted to electrophoresis. Proteins were then transferred on a nitrocellulose membrane and an overnight incubation was performed at 4° with specific primary antibodies for each transcription factor (1/2000 Myt1, 1/2500 Olig2, 1/500 Sox17) together with antibodies against GAPDH (1/2000 for Myt1), or β‐tubulin (1/1000 for Olig2 and Sox17). Incubation was further performed at room temperature with fluorescent secondary antibodies. Images were then acquired using chemiluminescent darkroom development technique. Quantification was done by densitometry of Western Blot bands using Image J software (NIH).

### 2.4 Statistical analysis

Data were processed using commercially available software GraphPad Prism 7.0 (GraphPad Inc, San Diego, CA). When the Shapiro‐Wilk test was applied on transcription factor mRNA and protein levels, *P* values were larger than 0.05 in all groups and we therefore assume a normal distribution. Both data points and mean ± standard error of the mean (SEM) are shown in graphs. One‐way ANOVA (analysis of variance) followed by Fisher's LSD (for mRNA levels) or Tukey's multiple comparisons (for protein levels) *post hoc* test was used to determine significance of differences among treatments. *P* values < 0.05 were considered statistically significant.

## 3 RESULTS

### 3.1 Effects of NESTORONE and E2 on transcription factor mRNA levels

**Figure 1:**
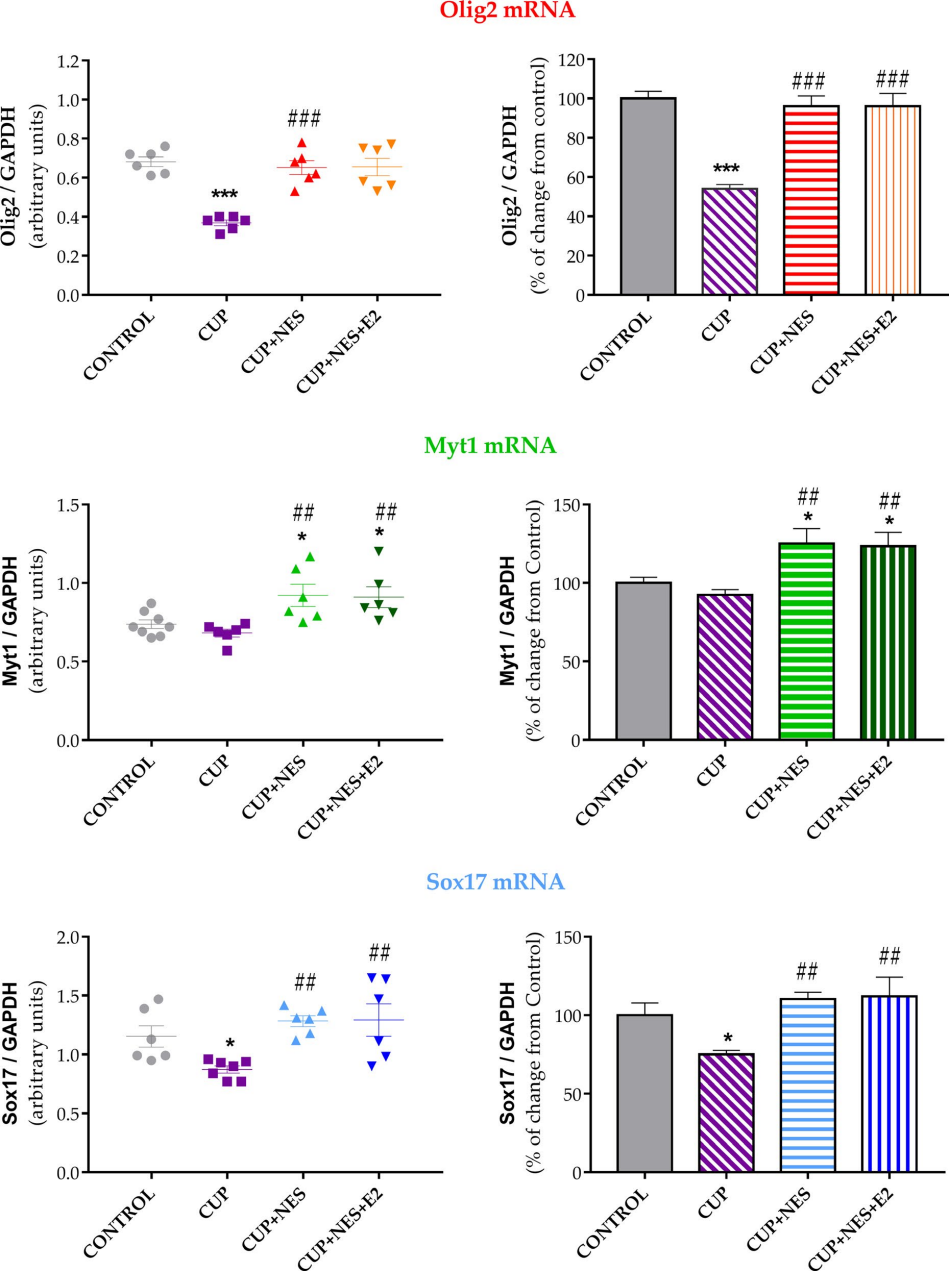
Effects of NES alone and combined with E2 on Myt1, Olig2, and Sox17 mRNA levels. The transcripts levels of the three myelin transcription factors were determined in the corpus callosum of female mice following 12‐week intoxication by CUP and a further 1‐week treatment with NES (CUP + NES 8 µg/d) or NES + E2 (CUP + NES+E2 1 µg/d). Data are expressed as individual values and mean ± SEM (Olig2 n = 6, Myt1 n = 6‐8, Sox17 n = 6‐7 per group). They were analyzed by one‐way ANOVA followed by Fisher's LSD *post hoc* test with significance placed at *P* < 0.05. *P < 0.05 and ***P < 0.001 vs CONTROL group; #*P* < 0.05, ##*P* < 0.01 and ###*P* < 0.001 vs CUP group

We first investigated the effects of a 1‐week treatment with 8 µg of NES/d and co‐administration of 1 µg E2/d following CUP diet on transcription factor mRNA levels. One‐way ANOVA indicated an overall significant effect of treatment on mRNA levels for Olig2 [F(3,20)] = 21.36, *P* < 0.0001, n = 6], Myt1 [F(3,22)] = 5.98, *P* = 0.0038, n = 6‐8], and Sox‐17 [F(3,21)] = 5.80, *P* = 0.0047, n = 6‐7]. We observed that the significant decrease in Olig2 mRNA levels after CUP treatment (by 46%, *P* < 0.0001) was restored to control levels by NES treatment alone (*P* < 0.001) and combined with E2 (*P* < 0.001) (Figure 1A). E2 co‐treatment did not change the effect of NES on Olig2 mRNA (CUP + NES vs CUP + NES + E2 *P* = 0.9420) (Figure 1A). In contrast, after CUP diet NES treatment alone or with E2 significantly stimulated Myt1 mRNA levels (*P* = 0.0029 and *P* = 0.0042, respectively; Figure 1B). An enhancement of ~25% above the control value was found significantly different in both cases (CONTROL vs. CUP + NES *P* = 0.0116, CONTROL vs CUP + NES + E2 *P* = 0.0171, respectively) (Figure 1B). E2 co‐treatment did not change the effect of NES on Myt1 mRNA (CUP + NES vs CUP + NES + E2 *P* = 0.8719). Sox17 mRNA levels were found to be significantly reduced by CUP (~25%, *P* = 0.0252) and significantly increased by NES treatment alone (*P* = 0.0020) or with E2 (*P* = 0.0017) (Figure 1C). E2 treatment did not significantly modify the increase in Sox‐17 mRNA levels induced by NES (*P* = 0.9457). For both treatments, an increase of ~10%‐12% above the control value was found but not statistically significant.

### 3.2 Effects of NESTORONE and E2 on transcription factor protein levels

**Figure 2:**
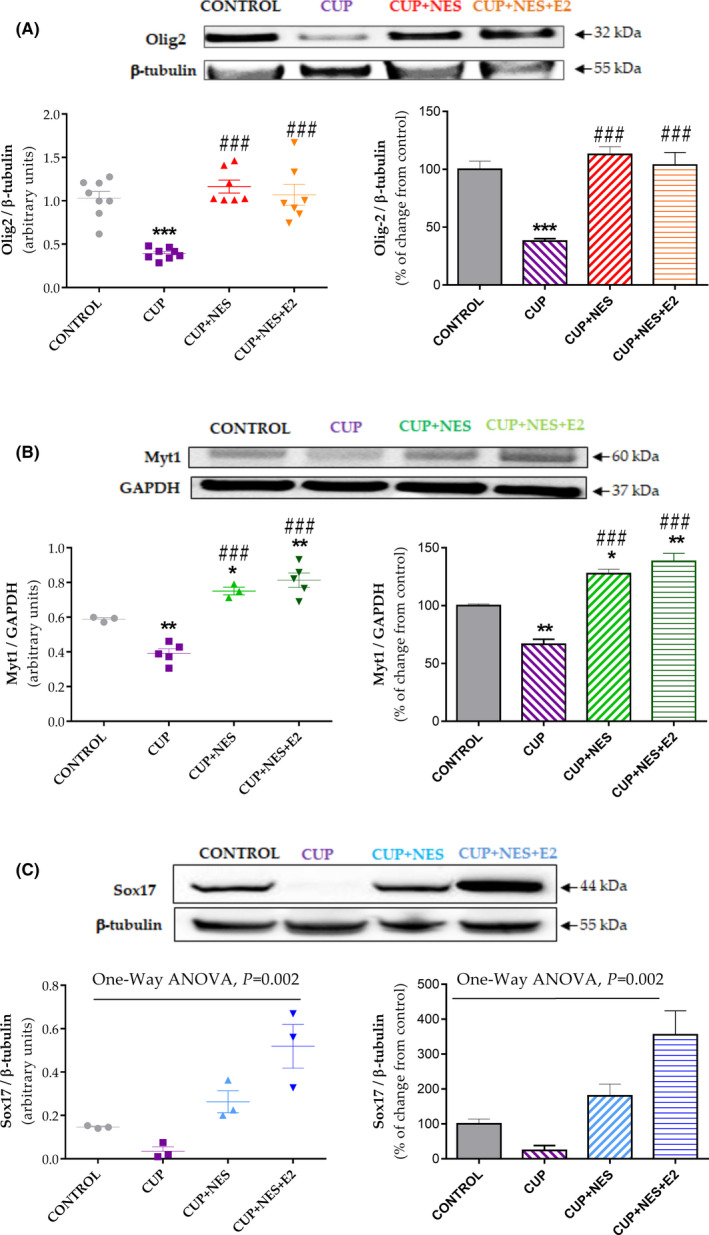
Effects of NES alone and combined with E2 on Myt1, Olig2, and Sox17 protein levels. The levels of the three transcription factors, Olig2 (A), Myt1 (B), and Sox17 (C), were determined in the corpus callosum of female mice following 12‐week intoxication by CUP and a further 3‐week treatment with NES (CUP + NES 8 µg/d) or NES + E2 (CUP + NES+E2 1 µg/d). Data are expressed as individual values and mean ± SEM (Olig2 n = 7‐8, Myt1 n = 3‐5; Sox17 n = 3 per group). They were analyzed by one‐way ANOVA followed by Tukey's multiple comparisons *post hoc* test with significance placed at *P* < 0.05. *P < 0.05 and **P < 0.01 vs CONTROL group; ##*P* < 0.01 and ###*P* < 0.001 vs CUP group

We then analyzed Olig2, Myt1, and Sox17 protein levels by Western blot after a 3‐week treatment with NES after CUP diet, in the presence or absence of E2. One‐way ANOVA also indicated a significant effect of treatment on protein levels for Olig2 [F(3,26)] = 21, *P* < 0.0001, n = 7‐8], Myt‐1 [F(3,12)] = 39, *P* < 0.0001, n = 3‐5], and Sox‐17 [F(3,8)] = 13, *P* = 0.0018, n = 3]. The levels of Olig2 and Myt‐1 significantly decreased in the CUP‐treated animals (by 62% for Olig2 *P* < 0.0001 and by 33% for Myt1 *P* = 0.0065) as compared to controls (Figure 2A and B). NES treatment significantly stimulated the synthesis of both transcription factors. A strong and significant enhancement was observed for Olig2 after NES treatment alone (3‐fold increase vs CUP, *P* < 0.0001) and NES with E2 (3‐fold increase vs CUP, *P* < 0.0001) (Figure 2B). Similarly, Myt‐1 levels increased significantly in mice treated with NES treatment alone (2‐fold increase vs CUP, *P* < 0.0001) and NES + E2 (2‐fold increase vs CUP, *P* < 0.0001). There was no significant difference between the two groups (*P* = 0.5687) but a significant enhancement of 28% (*P* = 0.043) and 38% (*P* = 0.002) above the CONTROL values, respectively. Finally, following CUP diet Sox‐17 levels were decreased ~4 fold compared to controls, increased ~7 fold by NES treatment alone and ~15 fold when combined with E2 (Figure 2C). The potentiation of NES effect by E2 on Sox17 levels was close to significance (*P* = 0.053). This augmentation was about 2 fold in CUP + NES‐treated mice and 4 fold in CUP + NES + E2 mice (*P* = 0.008) as compared to controls (Figure 2C).

## 4 DISCUSSION

Despite having achieved milestones at developing disease modifying therapies (DMT)^10^ that halt the progression of diseases like MS, there is a paucity of directed therapies to repair or regenerate myelin.^11^ Therefore, there is a need to understand mechanisms of remyelination and causes of remyelination failure to be able to better design strategies for enhancing remyelination. At the MS disease initiation, endogenous remyelination partly occurs after relapses but, with time, remyelination becomes less and less efficient. Usual treatment of MS with DMT reduces the number of relapses but does not act on myelin regeneration. One of the most effective ways to favor neuroprotection and limit disability would be to also enhance remyelination.

We had previously observed that after CUP‐induced demyelination for 12 weeks, remyelination did not spontaneously occur, whereas NES stimulated myelin repair through the production of myelin proteins such as Myelin Basic Protein and PLP.^1^ NES 3‐week treatment stimulated the recruitment of oligodendrocyte progenitor cells, the replenishment of mature oligodendrocytes, and myelin regeneration in the female mouse brain. Also, while demyelination in response to cuprizone was less marked in corpus callosum than in cerebral cortex, remyelination appeared earlier in the former.

Here, we show that NES restored or strongly enhanced the production of Myt1, Olig2, and Sox17, three transcription factors involved in myelin synthesis. The increase in production of these transcription factors in the corpus callosum appeared as early as after one week of NES administration. This observed increase in myelin regeneration appears as one of the possible mechanisms of myelin repair after demyelination in the rodent model.

To promote myelin repair, two research strategies can be adopted^11^: antagonizing signaling pathways which exert a negative regulatory role on OL maturation and myelination, such as the Lingo‐1,^12^ by antibodies, or blocking the muscarinic receptors by antagonists, like Benztropine.^13^ The second approach deals to stimulate OL differentiation. Most compounds tested for regeneration of myelin have been tested on OPC lines, for example Clobetasol, a glucocorticoid or Miconazole, an antifungal. However, these drugs were first tested in vivo in EAE^14^ and appeared promising, provided sufficient OPC persist in patients after relapses. Nestorone induced a positive effect both in CUP and EAE models.^15^ Further studies are required to clarify the mechanisms by which enhancement of Olig2 and Myt1 by NES in the CNS govern the production of OL cell lineage.

In conclusion, NES alone or combined with E2 favors remyelination in a rodent model of demyelination and increases the production of Myt1, Olig2, and Sox17, three transcription factors involved in myelin synthesis. Should this myelin repair mechanism be confirmed in humans, NES administration to MS patients, and coupled with E2 in women or testosterone in men, could be an efficient adjunct therapy to disease modifying agents. Translational studies in this direction are being prepared.

## CONFLICT OF INTEREST

RSW is an employee of the Population Council, a not‐for‐profit organization that developed Nestorone for contraception. She received a research grant from the National Institute of Child Health and Human Development (NICHD) from the National institute of Health (NIH) as Project Director of a Contraception research center U54 HD 29,990. RSW, MS, and MEE are co‐inventors on a patent on “Neuroprotection and myelin repair using Nestorone ®”. US 10,052,335 B. YA and MR report no conflict of interest.

## Supporting information

Fig S1

## Data Availability

The data that support the findings of this study are available from the corresponding author upon reasonable request.
