# Supplementary figures and images for: Nestorone®, a 19nor‐progesterone derivative boosts remyelination in an animal model of demyelination
Source: CNS Neurosci Ther. 2020 Dec 24;27(4):464–9. doi: 10.1111/cns.13538 (PMC7941173; doi:10.1111/cns.13538)

Figure 2 - blot A

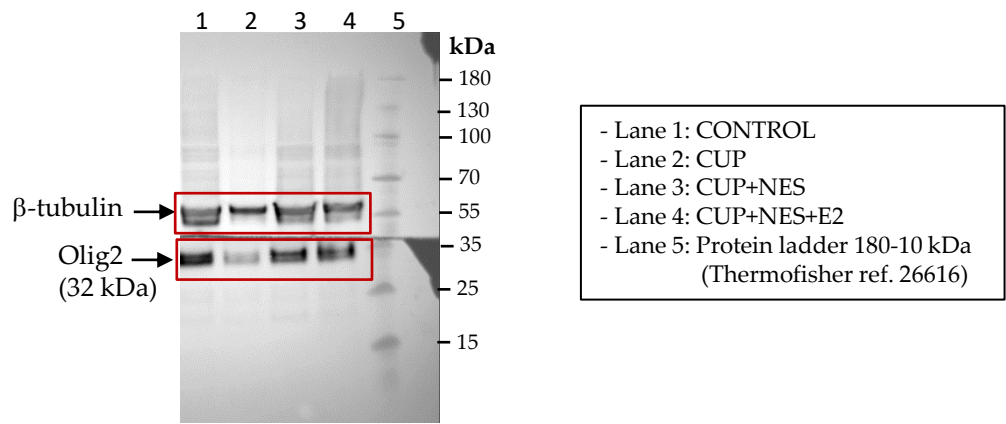

Figure 2 - blot B

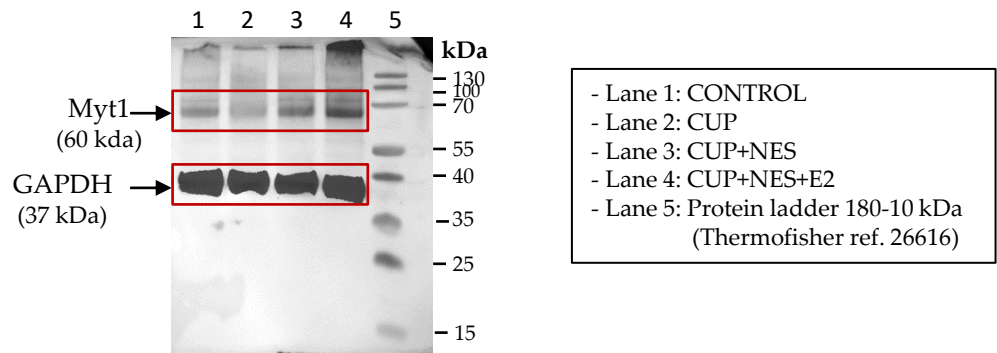

Figure 2 - blot C

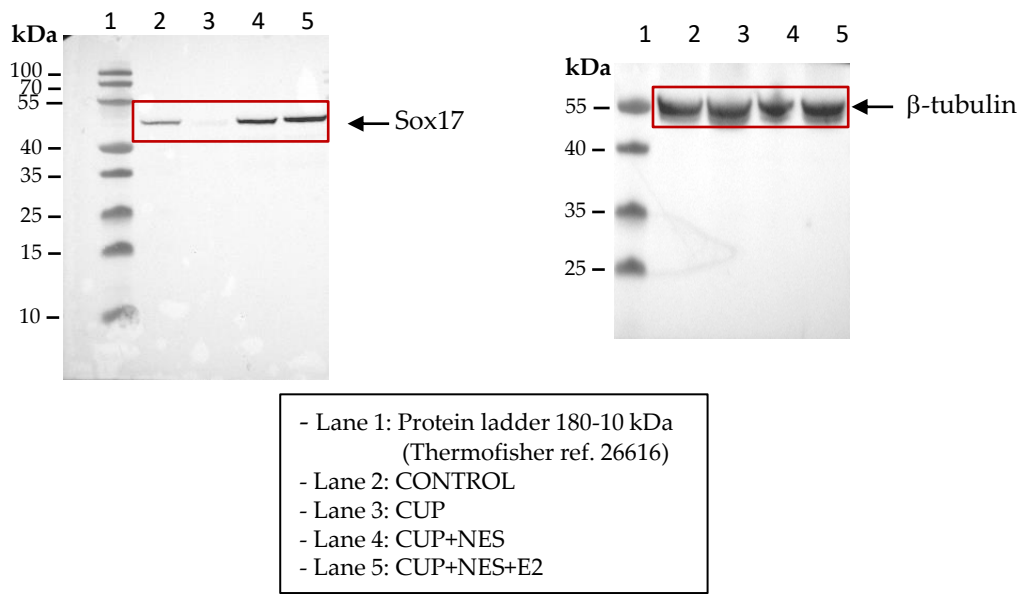

Supplement: Supplementary file 1 — Fig S1 [file CNS-27-464-s001.pdf]
